# Vaccinium Crassifolium Aut Repens

**Published:** 1880-06

**Authors:** 


### VACCINIUM CRASSIFOLIUM AUT REPENS.

Dr. E. A. Anderson, of Wilmington, in the North Carolina *Medical Journal* directs attention to this remedy as a diuretic in obstinate cases of dropsy from hepatic or cardiac disease in patients, broken down by intemperance and innutrition, or the result of poverty, vice and old age. It is a creeper growing in low, upland Savannahs, in moist, damp places, and in the margin of ditches, and closely resembles the uva-ursi of commerce. It is used in infusion or decoction. Its diuretic properties may be increased, and a very pleasant sub-acid beverage prepared by using cream of tartar, loaf sugar and lemon with ice. Dr. Anderson reports several cases in which he has observed marked effects from the use of this remedy, and also gives the endorsement of medical men who have obtained satisfactory results in cases of dropsical effusion. The vaccinium can be obtained by writing to John K. McIlhenny, druggist, Wilmington, N. C.

